# Attitude towards and Readiness for Interprofessional Education in Medical and Nursing Students of Bern

**DOI:** 10.3205/zma001072

**Published:** 2016-11-15

**Authors:** Ulrich Woermann, Lena Weltsch, Alexandra Kunz, Daniel Stricker, Sissel Guttormsen

**Affiliations:** 1 University of Bern, Institute for Medical Education IML, Education and Media Unit AUM, Bern, Switzerland; 2 Bern, Switzerland; 3 University of Bern, Institute for Psychology, Bern, Switzerland; 4 University of Bern, Institute for Medical Education IML, Bern, Switzerland

**Keywords:** Medical education, interprofessional learning, survey, medical study, nursing study

## Abstract

**Objectives: **Interprofessional collaboration is becoming increasingly important in health care for various reasons. Interprofessional Education (IPE) can provide a basis for this.

The aim of our study was to find out how medical (MS) and nursing students (NS) think about their own and other professions, what they know about each other, how strong their willingness to embrace IPE is, and what forms of IPE they deem useful.

**Methodology: **Seven IPE experts rated the two measuring instruments, Readiness for Interprofessional Learning Scale RIPLS, and Interdisciplinary Education Perception Scale IEPS in terms of relevance of the items, and the quality of translation into German. Nine RIPLS items and 13 IEPS items were considered content-valid. All MS of the University of Bern and NS of the two Bernese educational institutions for nursing were invited to the online survey in the fall of 2014 by email.

**Results: **498 (254 MS, 244 NS) of the 2374 invited students completely filled in the questionnaire (21%).

The results of the reduced RIPLS allowed no conclusive statements. When assessing their own occupational group in the IEPS, the MS attributed "competence and autonomy" to themselves significantly more frequently, while to the NS, the same was true for the item, "actual cooperation". MS know significantly less about the training of other health professionals. NS show a significantly higher willingness to embrace IPE. Teaching ethics, communication, team training, and clinical skills are deemed suitable for IPE by both groups. From the comments it appears that in both groups a majority welcomes IPE; however, the various arguments had different prevalence in both groups. Both groups fear that IPE leads to heightened stress during the study. A subgroup of MS fears a lowering of academic level.

**Conclusion: **The results of this survey of Bernese MS and NS concerning IPE provide important information for the planning and implementation of IPE. Important steps in the introduction of IPE will be a clear justification and the definition of its objectives. These must be explicitly communicated to all students.

## Zusammenfassung

**Zielsetzung: **Interprofessionelle Zusammenarbeit wird im Gesundheitswesen aus verschiedenen Gründen immer wichtiger. Interprofessional Education (IPE) kann eine Basis hierfür schaffen.

Ziel unserer Studie war zu erfahren, wie Medizin- (MS) und Pflegestudierende (PS) über ihre eigene und andere Berufsgruppen denken, was sie voneinander wissen, wie ihre Bereitschaft zu IPE aussieht und welche Formen von IPE sie für sinnvoll halten.

**Methodik: **Sieben IPE- Expertinnen und- Experten beurteilten die zwei Messinstrumente Readiness for Interprofessional Learning Scale RIPLS und Interdisciplinary Education Perception Scale IEPS bezüglich Relevanz der Items sowie die Qualität der Übersetzung ins Deutsche. Neun Items der RIPLS und 13 Items der IEPS wurden als inhaltlich valide erachtet.

Alle MS der Universität Bern und PS der zwei Berner Pflege Bildungsinstitutionen wurden im Herbst 2014 per E-Mail zur Online-Umfrage eingeladen.

**Ergebnisse: **498 (254 MS, 244 PS) der 2374 eingeladenen Studierenden füllten den Fragebogen vollständig aus (21%).

Die Resultate der reduzierten RIPLS erlaubten keine schlüssigen Aussagen. Bei der Einschätzung der eigenen Berufsgruppe in der IEPS schrieben sich die MS signifikant häufiger „Kompetenz und Autonomie“ zu, die PS hingegen signifikant häufiger „Tatsächliche Kooperation“. MS wissen signifikant weniger über die Ausbildung anderer Gesundheitsberufe. Bei PS besteht signifikant höhere Bereitschaft zu IPE. Lehre zu Ethik, Kommunikation, Team-Training und Clinical Skills werden von beiden Gruppen als für IPE geeignet erachtet.

Aus den Kommentaren geht hervor, dass beide Gruppen IPE mehrheitlich begrüßen, wobei die verschiedenen Argumente unterschiedlich häufig genannt werden. Beide Gruppen fürchten, dass IPE zu vermehrter Belastung im Studium führt. Eine Untergruppe der MS befürchtet eine Senkung des akademischen Niveaus.

**Schlussfolgerung:** Die Resultate dieser Umfrage zu IPE bei Berner MS und PS liefern wichtige Informationen für die Planung und Durchführung von IPE. Wichtige Schritte bei der Einführung von IPE werden eine klare Begründung sowie die Definition ihrer Ziele sein. Diese müssen explizit allen Studierenden kommuniziert werden.

## 1. Introduction

On the one hand, the increasing specialization in medicine keeps producing new occupations; on the other hand, increasing numbers of chronically ill patients require that non-physician health professionals be ever more involved in their medical care. These two trends make the issues of interprofessional collaboration, i.e. cooperation of different professional groups in the workplace, and interprofessional education (IPE), i.e. learning from, with and about other occupational groups [http://caipe.org.uk/about-us/defining-ipe/ last visited on 29 Oct., 2015], ones of increasing importance. In addition, the increasing personnel and resource scarcity in healthcare adds to the urgency to organize work more efficiently. These changes require that all healthcare professionals know more about and are able to better communicate with each other to ensure optimal patient care. Bhutta et al. are looking, in this context, “to overcome professional silos” [1]. Both the World Health Organization WHO and the Swiss Federal Office of Public Health have published corresponding recommendations for IPE [2], [3] despite the fact that up until now the evidence that IPE helps improve patient care has been limited [4]. When it comes to planning IPE events, the focus is usually on analyzing the faculty’s perspective [5]. Rarely is the target audience of IPE, the students, asked about their attitude towards IPE or knowledge about other health professionals [6]. This information can, however, be very useful for the planning of IPE events and their success.

We wanted to find out through a survey what medical (MS) and nursing students (NS) think about their own and other professions, what they know about each other, to what degree they are willing to embrace IPE, and what forms of IPE they deem useful. The results of the survey shall be incorporated in future planning of IPE in the Bern area. The invitees to the survey in the fall of 2014 were: all MS of the University of Bern; all NS at the Bern University of applied Sciences Health (Bachelor course, FH) and the Bern College of Higher Education in Nursing (Bildungszentrum Pflege) (Diploma course, HF). 

## 2. Method

### 2.1 Selection, translation and validation of existing measuring instruments

We selected three measuring instruments for learning about the attitude towards other occupational groups and for detecting the readiness for IPE. Our selection criteria were the free availability of the questionnaire as well as its commonness in the literature. The selected instruments were: the Readiness of Healthcare Students for Interprofessional Learning Scale RIPLS [7], the Interdisciplinary Education Perception Scale IEPS [8], and the Attitudes to Health Professionals Questionnaire AHPQ [9].

The two first authors translated from English into German the three measuring instruments which comprised a total of 57 questions. With this number of questions, there was the obvious risk that some students would terminate the survey prematurely due to “evaluation fatigue”, an observation made at all three educational centers. Therefore, we reduced the number of questions after we had a group of experts assess their relevance. We found five female and two male experts who were native German speakers, and who – having been involved in the planning of IPE events either as tutors or as professionals – were willing to assess the relevance of each item. Based on a scale from 1=“very relevant” to 6=“not relevant”, they were to assign a value to each item. Items that scored an average of 2 points or less were included in our survey. Since the majority of the mean values were less than 3, we set the threshold value to 2 to achieve a clear discrimination. Also, we had the experts assess the translation of each item. The English wording and the German translation were displayed side by side, and comments and amendments could be made. Out of the 19 RIPLS items, 9 were considered by the expert group as relevant (see Attachment 1, item a). Out of the 18 IEPS items, 13 classified as relevant (see Attachment 1, item b). None of the 20 AHPQ items reached the required=2.0, and since the group of experts also criticized the unclear wording of the questions in this instrument, we decided not to include the AHPQ in the survey.

The analysis of the results of RIPLS and IEPS was performed using factor analysis with Varimax rotation and extraction factors according to the Kaiser criterion (Eigenvalue>1.0) [10]. To compare the two professional groups, the NS of FH and HF, respectively, were grouped together because no significant differences in the response patterns could be found between those two groups.

#### 2.2 Added proprietary questions

##### 2.2.1 Knowledge about one‘s own and other professional groups

To completely cover our research target, we formulated questions concerning further topics not covered by the existing instruments. These questions dealt with the prerequisites (max. score: 34), duration (max. score: 14), and certificate (max. score: 18) of 14 different professional trainings in healthcare in Switzerland (see Attachment 2) [http://caipe.org.uk/resources/defining-ipe/ last visited on 29 Oct., 2015]. Overall, this amounted to a possible score of 66. However, some experts found these added questions to be of little relevance, but formulating alternative, unambiguous questions such as about competence areas was difficult to them. Therefore, we retained these questions, but considered them only as indicators of knowledge about other professional groups. When processing the answers to these questions, we looked at the two groups of NS separately in consideration of the fact that their training differs greatly in the three issues mentioned above.

##### 2.2.2 Proprietary questions about readiness for IPE

To capture the readiness for IPE, six questions were formulated in accordance with “AMEE Guide No. 87” (13). These questions, too, were assessed by the expert group regarding formulation and meaningfulness. These questions are listed in Table 1 (Tab. 1) along their options.

Statistical analysis was performed using factor analysis and chi-squared test. 

#### 2.3 Free comments

With three questions, students were asked to formulate reflections on their answers as free text (see Table 1 (Tab. 1)). These comments were checked for favorable and unfavorable arguments. Furthermore, the arguments were organized into categories resulting from text analysis. 

#### 2.4 Comprehension check

As a last step of quality control, a selection of students was asked if they understood all questions of the questionnaire. Of each education center, three students per study year were randomly selected (a total of 36) to assess their comprehension of the items in the questionnaire made available online, and to comment if not fully understood. These 36 students were then excluded from the survey proper.

#### 2.5 Demographic data

In addition to age, gender, field of study, and year, participants were asked if they had already completed vocational training or a study, if they had been aware of IPE before this survey, and if so, in what context, and whether they had already been involved in an IPE project. 

#### 2.6 Assessment of responsibility

Based on the final version of the survey a "Request for assessment of responsibility" was filed at the Cantonal Ethics Committee of Bern (KEK). The subsequent presidential decree noted that the KEK was not responsible for this project. For the conduction of the survey, this meant that it was not subject to authorization, as it was not covered by the Human Research Act, art. 2, para. 1.

#### 2.7 Conduction of the survey

The survey was put online with SurveyMonkey®. The students were invited by e-mail in mid-October, 2014, by the respective administration offices of the three training centers. Four weeks after the first invitation a reminder was sent. The period from mid-October to late November was chosen because in none of the three schools examinations took place during that time; therefore, a higher response rate was to be expected. At the end of November, 2014, the survey was closed.

## 3. Results

### 3.1 Response rate

A total of 2374 invitations were sent out (University: 1317, FH: 268, HF: 789). Of the 620 students who started the survey, 122 (20%) terminated it prematurely. Only the data of 498 completed questionnaires were included in the analysis. The response rate was thus 21% (University: 19.3%, FH: 17.9%. HF: 24.8%).

#### 3.2 Demographic data

The gender and age distribution in all three groups corresponded to the distribution at each educational institution. In all training years, a similar MS number responded, while in NS, only a few first-year students responded. 

#### 3.3 RIPLS

In the factor analysis of RIPLS reduced to 9 items, only one factor could be found. The total score yielded no conclusive results. Although there are significant differences between MS and NS, these account for only just 2.7% of the variance. Therefore, we consider the results of the reduced RIPLS to be unusable. 

#### 3.4 IEPS

Based on factor analysis and according to the scree plot three factors could be determined which explain 55% of the variance. The following subscores could be defined (see Attachment 1, item b concerning numbering of items):

(Internal consistency Cronbach's alpha 0.76 for all 13 items; 0.59 for subscore 1, 0.435 for subscore 2, 0.85 for subscore 3)

- Competence and autonomy, items 1, 3, 4, 13
- Perception of the need for cooperation, items 5, 6
- Perception of the actual cooperation, items 2, 7, 8, 9, 10, 11, 12

The level of agreement with each item was expressed on the Likert scale (6=“strongly agree” to 1=“strongly disagree”).

The biggest difference between MS and NS was found in the subscore, "competence and autonomy" where MS (mv=4.82 women, 4.90 men) agreed to a significantly greater degree than NS (mv=4.30 women, 4.05 men). This effect explains 18% of the variance (F (1, 494)=111.820, p<0.001, Eta_p_^2^=0.185). Regarding the perception of the need for cooperation, no differences could be found. With actual cooperation, the agreement in NS (mv=4.76 women, 4.68 men) was significantly greater than in MS (mv = 4.15 women, 4.28 men). This explains 10% of the variance (F (1, 494)=54.607, p<0.001, Eta_p_^2^=0.100).

Across all participants there were no gender differences. With the subscore, "competence and autonomy" there was a significant difference between the male MS and the male NS (F (1, 494)=111.820, p<0.001, Eta_p_^2^=0.185). Whether students had heard of IPE or had even been involved in IPE had no significant effect on the results. 

**Figure 1:**
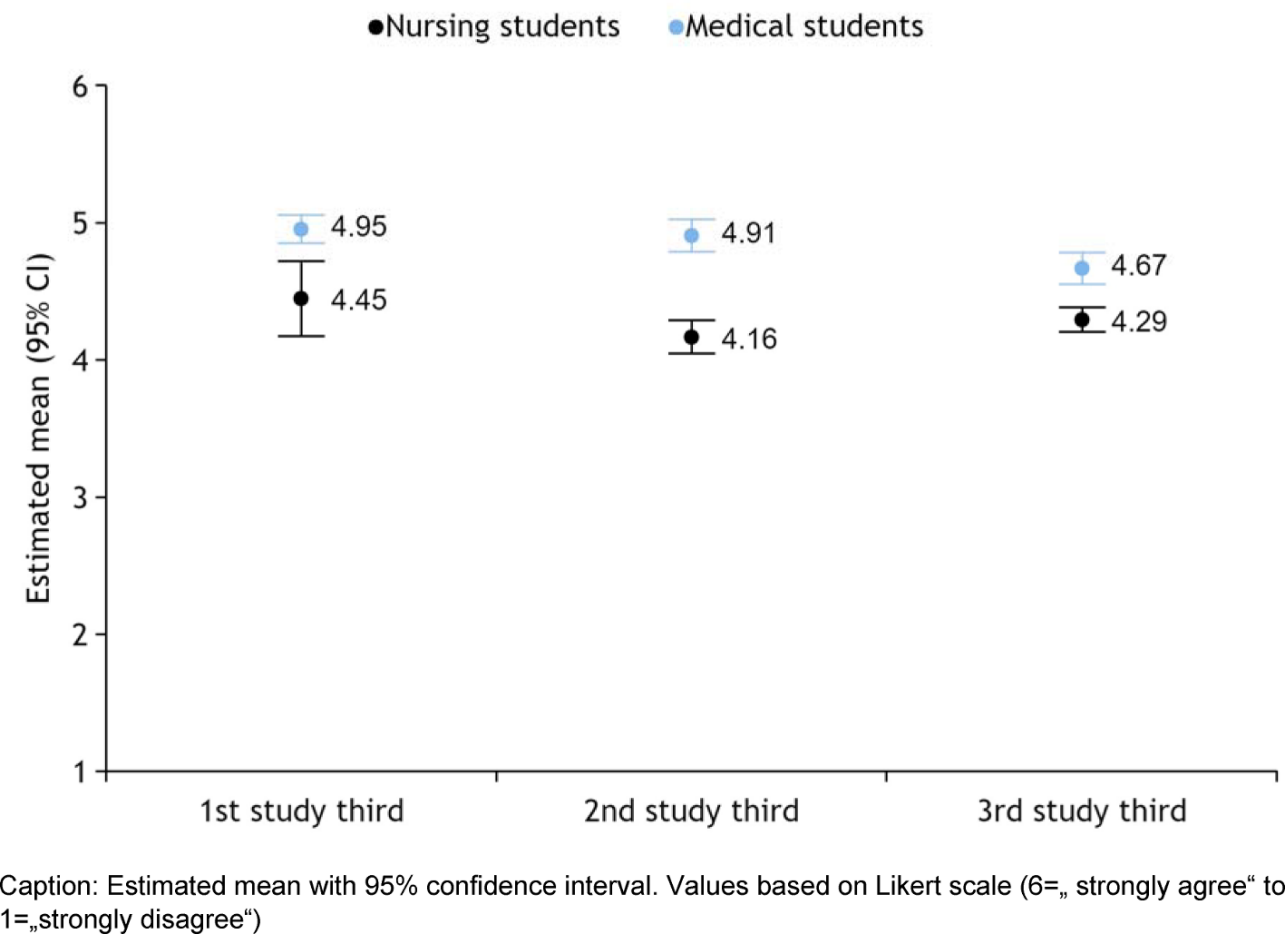
Advocacy of factor 1 „competence and autonomy” in IEPS

The academic year played only a significant role in the MS’ subscore, “competence and autonomy”. There, a decreasing approval across the study years was apparent (year 1: mv=4.96; year 2: mv=4.94; year 3: mv=4.85; year 4: mv=4.99; year 5: mv=4.7; year 6: mv=4.64; F (5, 248)=0.982, p=0.004, Eta_p_^2^=0.067). This was evident even when item 3 in MS was looked at alone (year 1: mv=4.2; year 2: mv=4.3; year 3: mv=4.25; year 4: mv=4.4; year 5: mv=4.1; year 6: mv=3.6; F (5, 248)=1.915, p=0.025, Etap2=0.050). In order to compare NS and MS to each other, we summarized the study years to study thirds. Here, too, only in MS in subscore 1, there was a significant decrease (F (2, 251)=8.096, p<0.001, Eta_p_^2^=0.061) (see Figure 1 (Fig. 1)).

#### 3.5 Proprietary questions

##### 3.5.1 Knowledge about one’s own and other professional groups

The total score differs significantly across the three groups (NS FH: mv=41.3; NS HF: mv=34.2; MS mv=22.7; p<0.001). It is striking that the MS very well know about their own training, but considerably less about nurses’ education. In the NS, the corresponding difference is significantly lower (see Table 2 (Tab. 2)).

NS FH have higher scores than MS and NS HF because they know considerably more about the training of midwives, physiotherapists and nutritionists. These professions, too, are taught at the University of Applied Sciences Health. 

##### 3.5.2 Readiness for IPE

**Figure 2:**
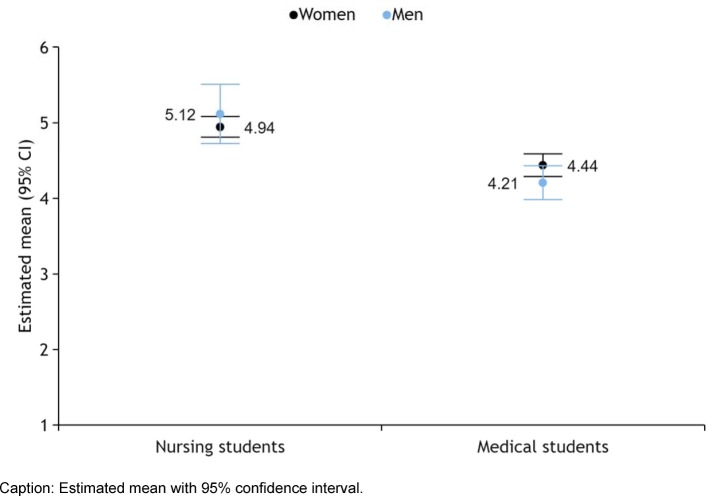
Advocacy of IPE events

Both MS and NS would mostly welcome the introduction of IPE events, but NS significantly more so (see Figure 2 (Fig. 2)). Also, NS would participate more if these events were optional (female NS: mv=4.41; male NS: mv=4.76; female MS: mv=4.21; male MS: mv=3.93; p<0.001). It is striking that the willingness to embrace IPE is higher in male NS than in female NS, while the opposite is true for MS.

**Figure 3:**
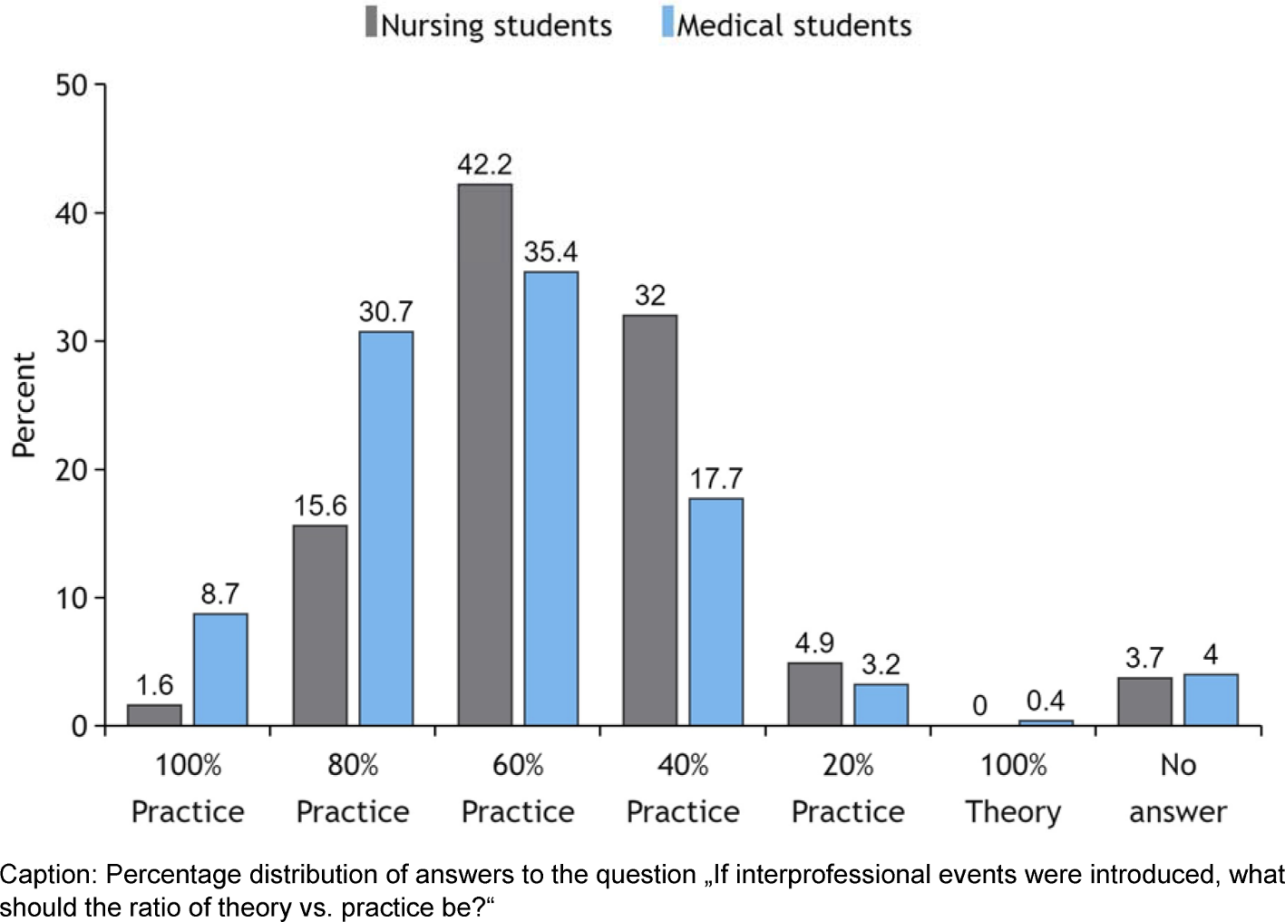
Proportion of theory vs. practice

The advocacy of education in interprofessional instead of single-professional groups is significantly higher in NS (interprofessional: 73% of NS; 54% of MS; single-professional: 12% of NS, 20% of MS; undecided: 13% of NS, 22% of MS; Chi^2^(3)=21.556, p<0.001). Regarding the proportion of theory vs. practice in IPE events, both groups put more emphasis on practice, but MS significantly more so (Chi^2^(6)=37.651, p<0.001) (see Figure 3 (Fig. 3)).

**Figure 4:**
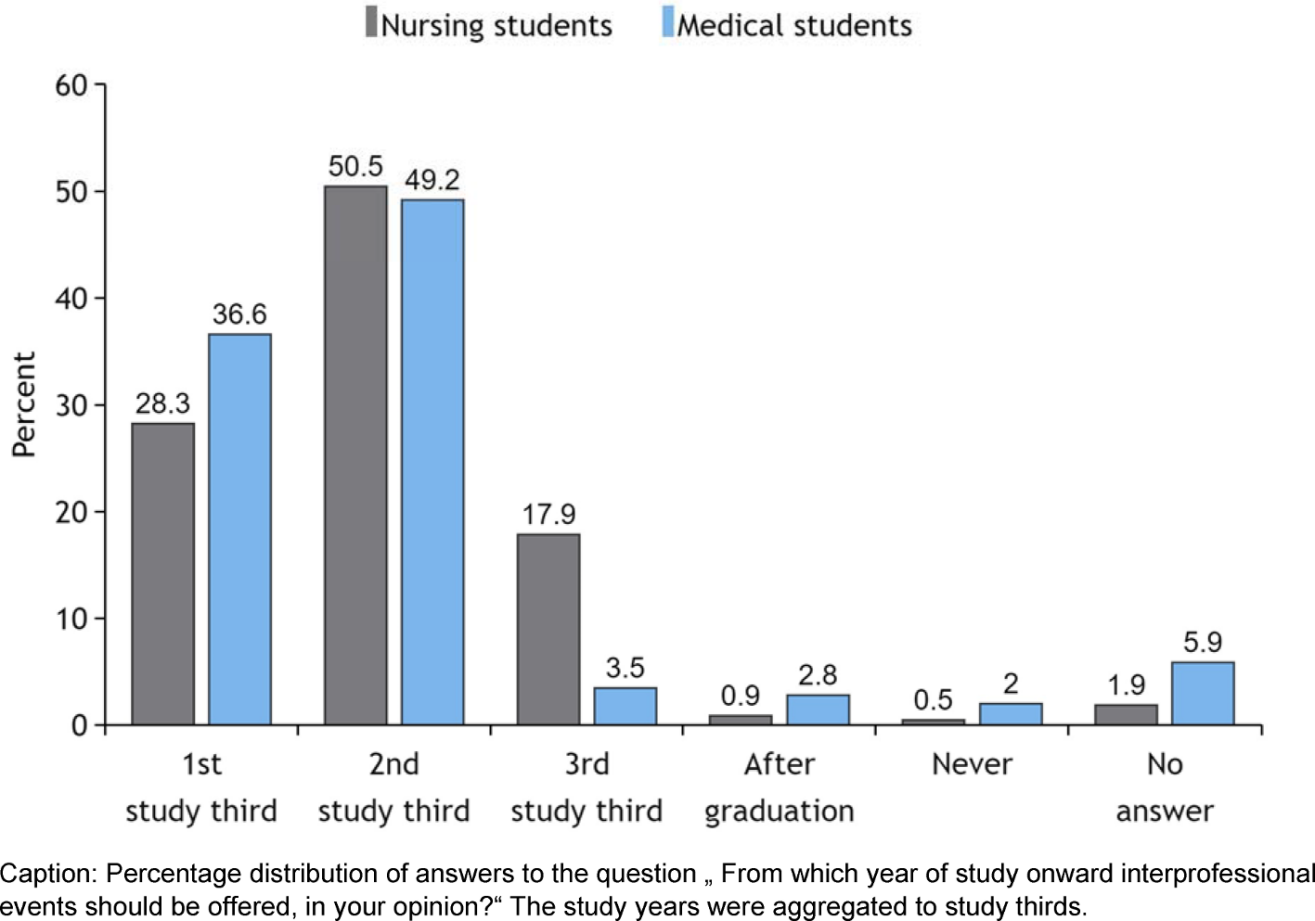
Start of IPE

When asked at what point should IPE be integrated into the study, both groups found the middle portion of the training most appropriate (see Figure 4 (Fig. 4)), followed by the first third.

For the answers given about appropriate topics for IPE a factor analysis was performed. Three factors could be defined which explain 64% of the variance. The subjects can be divided into the following three groups:

- Factor 1: Classical subjects (Cronbach's alpha 0.85), Items 1, 2, 6, 8, 11
- Factor 2: Communication & ethics (Cronbach's alpha 0.74), Items 4, 5, 7, 9, 10
- Factor 3: Clinical skills, item 3

**Figure 5:**
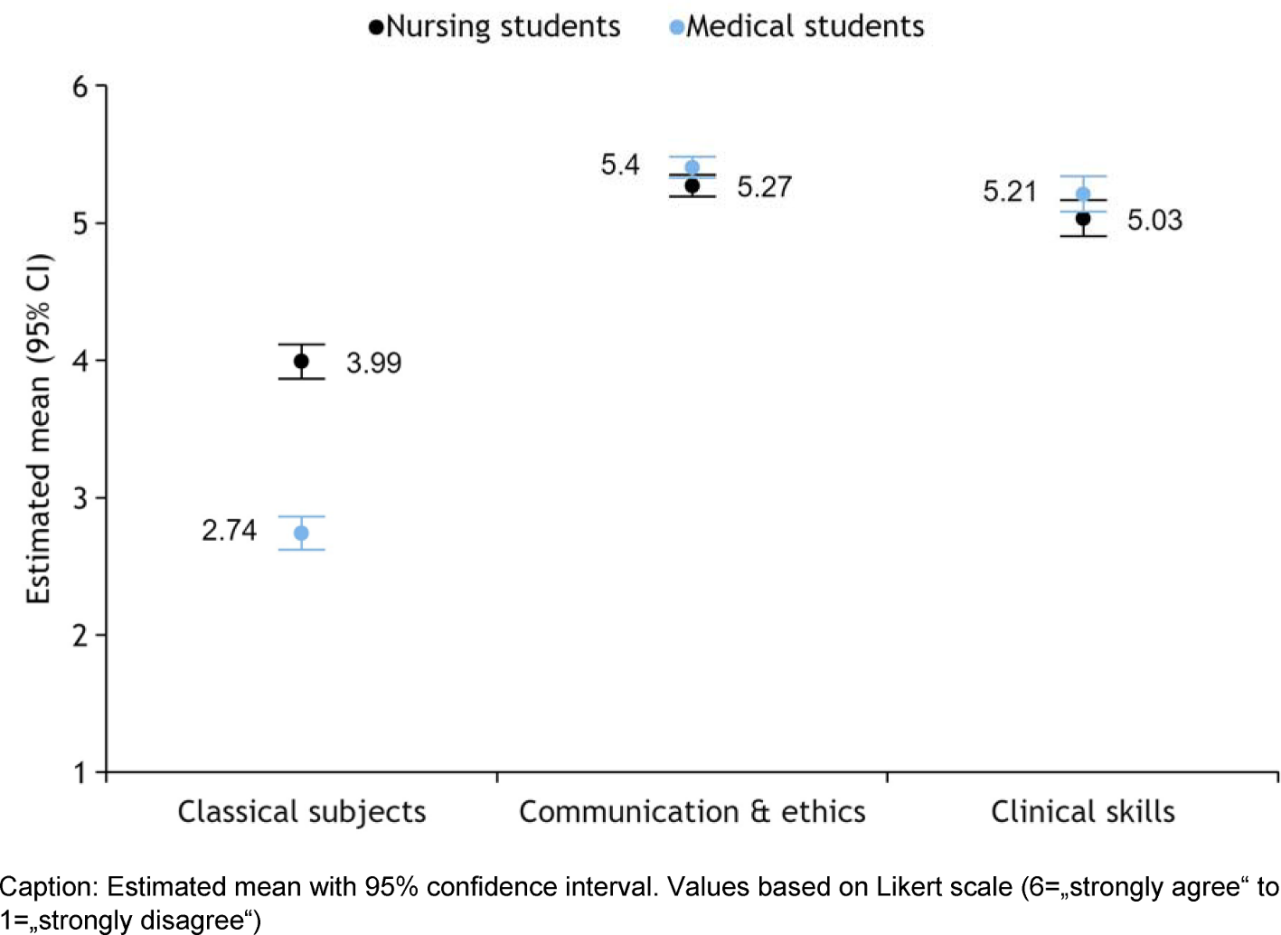
Appropriate topics for IPE

Only in factor 1 there was a significant difference. While both groups found classical subjects suitable for IPE to a limited extent only, MS think they are clearly less appropriate (see Figure 5 (Fig. 5)). Both groups clearly endorse factors 2 and 3.

##### 3.5.3 Free comments

498 participants entered a total of 696 comments (MS: 357; NS: 339) as a complement to the three questions. In analyzing the comments, the arguments were grouped into ‚advocating IPE‘ (MS: 349; NS: 371) and ‚dismissing IPE‘ (MS: 83; NS: 23). Further differentiation of arguments into additional categories deriving from the comments is shown in Table 3 (Tab. 3) below. 

In both groups favorable arguments outweighed unfavorable ones. However, the weighting of favorable arguments varied strongly. In the category, "conditional approval", demands were made regarding didactics, parity of the occupational groups, as well as a clear definition of the objectives of IPE. The highest number of negative arguments put forth in both groups concerned the question of participation in voluntary IPE events, but clearly more so in the MS group. The main negative argument given by both groups was lack of time/work overload. Additionally, the MS made a point of the difference in demands on knowledge and quality level.

## 4. Discussion

This online survey in Bernese MS and NS (FH and HF) on attitude towards and willingness to embrace IPE provided data as a basis for planning and designing IPE in Bern. Although the response rate of 21% may appear small, nearly 500 completed questionnaires do allow a reliable statistical analysis. Unlike Curran et al.’s observation of poorer response of physicians compared to nurses, the response rate of our MS was not worse than the one of the NS [5].

The results of the reduced RIPLS proved unusable. This must not be solely on account of the reduction of the items, but may also be due to the weakness of RIPLS itself [11], [12].

The IEPS proved to be a useful tool both in the validation by the expert group as well as in evaluating the results. Factor 1, “competence and autonomy” showed that the MS deemed their prospective profession to be endowed with more competence and autonomy than other health professions (see Figure 1 (Fig. 1)). This finding matches the results of Aase et al., who found in Norwegian MS and NS that traditional patterns still prevail in the perception of various health professions [13]. The fact that this self-assessment of MS decreases over the six years of study suggests that this perception diminishes by virtue of growing clinical experience. Coster et al. in their longitudinal study have made the observation that students of eight different health professions other than NS perceive their professional identity to decrease with growing clinical experience over time [6]. Professional identity, competence, and autonomy are categories that overlap. In NS, we observed no decline in factor 1 either. 

The difference in knowledge about other professional groups between MS and NS is very large. From several comments made it can be assumed that many MS are aware of this shortcoming. That MS clearly know less about the other health professions could be explained by their focusing mostly on their MD career, while the NS probably gave consideration to other health professions at the same level of education, too. In addition, NS FH learn their trade at the same school as midwives, physiotherapists and nutritionists and attend IPE events together. Also, prior to their study, many NS HF complete a training for health specialist. Even if one takes the knowledge about the prerequisites, duration and certificate of the training of 14 health professions as a mere indicator, the knowledge of MS about other health professions is still unacceptably low.

Both MS and NS would welcome part of the courses to be held interprofessionally (see Figure 2 (Fig. 2)). However, the attitude of NS towards IPE is considerably more positive; they are also more willing to participate in optional IPE events. This is a finding found again and again in the literature [5]. Reeves et al. provided a historical explanation for this phenomenon [14]. They argued that the medical profession was the first health profession that organized itself and for which entry barriers were set up to regulate access. However, it can generally be observed that professions higher-rated in terms of competence and hierarchy tend to distinguish themselves from lesser occupations, which is true for non-MD professions, too. MS advocate interprofessional settings for IPE less clearly than NS. Reeves et al. points out that single-professional settings are insufficient for promoting effective co-operation [14].

When it comes to define the relationship between theory and practice for IPE, MS favor practical settings more clearly than NS (see Figure 3 (Fig. 3)). 37% of NS favor a setting with an emphasis on theory. This reflects both the lack of practical clinical training often perceived by MS and the lack of theoretical training perceived by many NS. 

When asked about the best point in time for the IPE event, the middle third of study got the most votes, followed by the first third. Coster advocates starting IPE in the first year, as she has observed in her study that the readiness for IPE is highest in the first year, decreasing afterwards in all occupational groups except nursing [6]. Our data, too, reveal a trend towards decreasing acceptance of IPE, albeit not a significant one. The reason why the students prefer to begin with IPE only in the middle of study is probably because at the beginning they still feel insecure in their new role. At least, some of the free comments suggest this. 

Both NS and MS consider issues such as communication, teamwork, patient safety and clinical skills very suitable for IPE, in contrast to the “classical subjects”. That a classical subject like anatomy can be very well suited for IPE has been shown by a series of articles that have appeared lately [15], [16], [17]. However, the condition seems to be that these are practical courses, in which students can interact with each other. Reeves et al., too, indicate that IPE is most effective when interactive learning methods are used [14].

Although both groups in their comments judge IPE generally positively, they weight their arguments differently (see Table 3 (Tab. 3)). MS most often mentioned teamwork and cooperation, while for NS, expanding one’s horizon and acquiring additional knowledge is in the foreground. Lack of time and work overload are the main arguments against IPE put forth by both groups. A subgroup of MS reject IPE because they believe that great differences in knowledge and level make collaborative learning impossible or jeopardize their own learning success. In the literature we found no reports of similar comments. Better patient care or economic aspects appeared only three times in the comments, although these are two main arguments for IPE. It seems that students do not yet view themselves as players in healthcare. 

## 5. Limitations

Reducing the items of RIPLS and IEPS with the aim to obtain a higher response rate caused our results collected with these instruments to be comparable to other studies only to a limited degree. However, reduction of items of a survey is not an unusual approach, as McFayden et al. showed [18]. Also, not all best practice rules for the translation of instruments were applied, such as defined by the WHO.

The extent to which the data obtained from Bernese MS and NS can be generalized for the German-speaking countries and beyond is difficult to assess. Due to the relatively high homogeneity of German-speaking Switzerland it can be assumed that the results are representative at least for this region. Comparing our results with the international literature shows that other authors have obtained similar results [5]. As participation in the survey was voluntary, it is possible that especially proponents of IPE responded. However, the critical and sometimes, negative comments made rather contradict this assumption. But if really mainly proponents had participated, we would have to assume a more pronounced rejection of IPE by Bernese MS and NS. 

## 6. Conclusions

Our survey showed that both MS and NS have a mostly positive attitude towards IPE, but that there are significant differences in attitudes, expectations and knowledge about each other. These differences must be taken into account when planning IPE. Hence, the first step will be to justify the introduction of IPE and to define their objectives. These reasons and objectives must be explicitly communicated to the students.

The selection of topics and contents of IPE must be governed by these objectives while bearing in mind that students prefer practical contents. Didactic formats should be chosen which enable students to gather in groups with equal representation of MS and NS and to interact with each other, thereby diminishing bias and establishing mutual respect. IPE should be introduced no later than midpoint of the trainings. IPE has to be integrated into the respective curricula, without constituting an additional workload for the students in terms of time and subject matters. The challenge lies in either taking up in IPE issues that are already part of the curricula, or – by appropriate prioritization – in reducing other contents in favor of IPE.

## 7. Acknowledgements

A special thank you goes to Theres Scherrer of the Bern University of Applied Sciences Health and Claudia Schlegel from Bern College of Higher Education in Nursing, who made it possible that we could include the students of their schools in the poll.

Next we would like to thank the seven experts for taking the time to assess our translation and questions of the three measuring instruments and to asses and comment on our issues critically.

## Competing interests

The authors declare that they have no competing interests.

## Supplementary Material

Attachment 1: Assessment of relevance of RIPLS and IEPS items

Attachment 2: Prerequisites, duration, and certificate of 14 different studies/trainings

## Figures and Tables

**Table 1:**
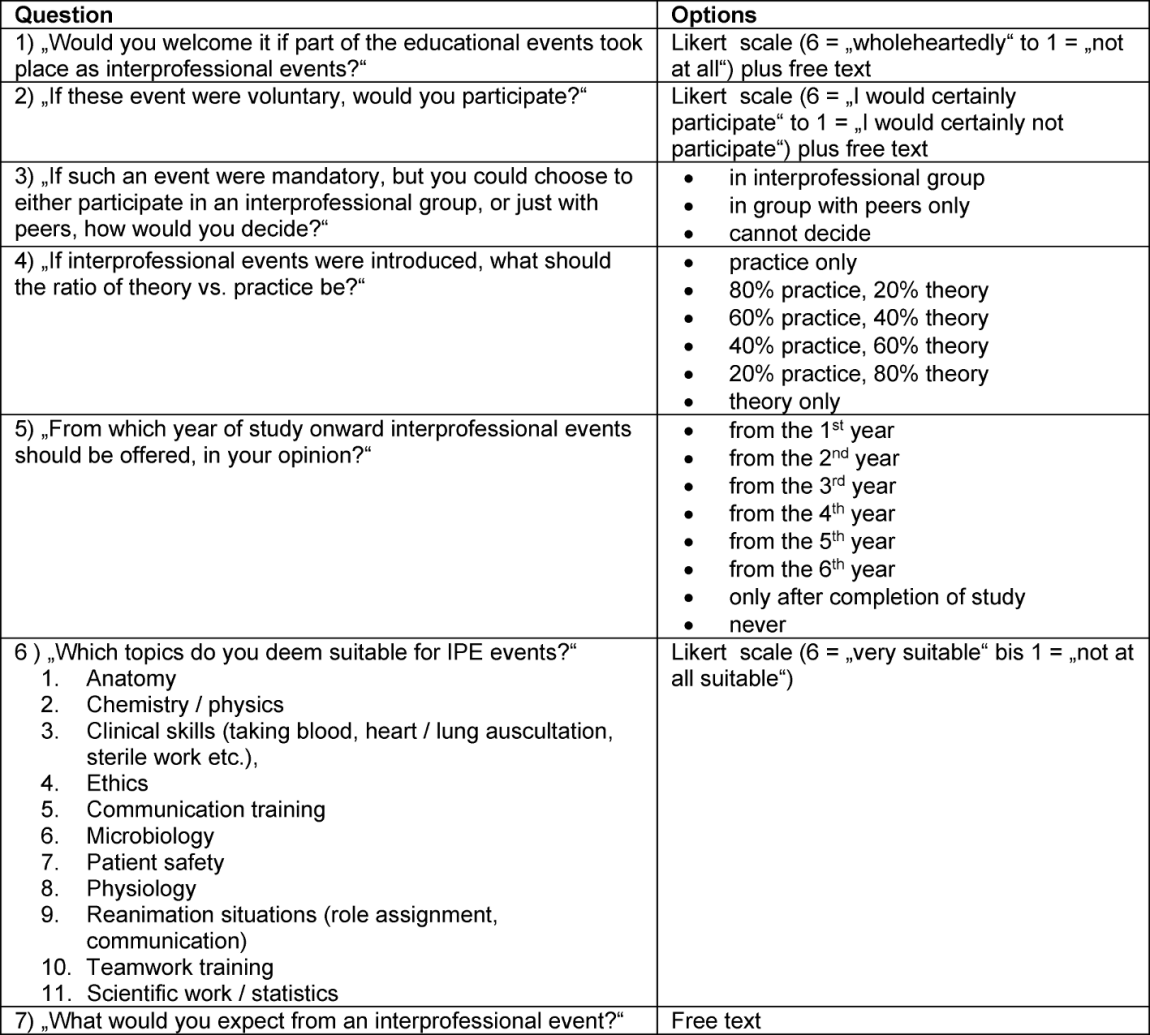
List of proprietary questions with options

**Table 2:**
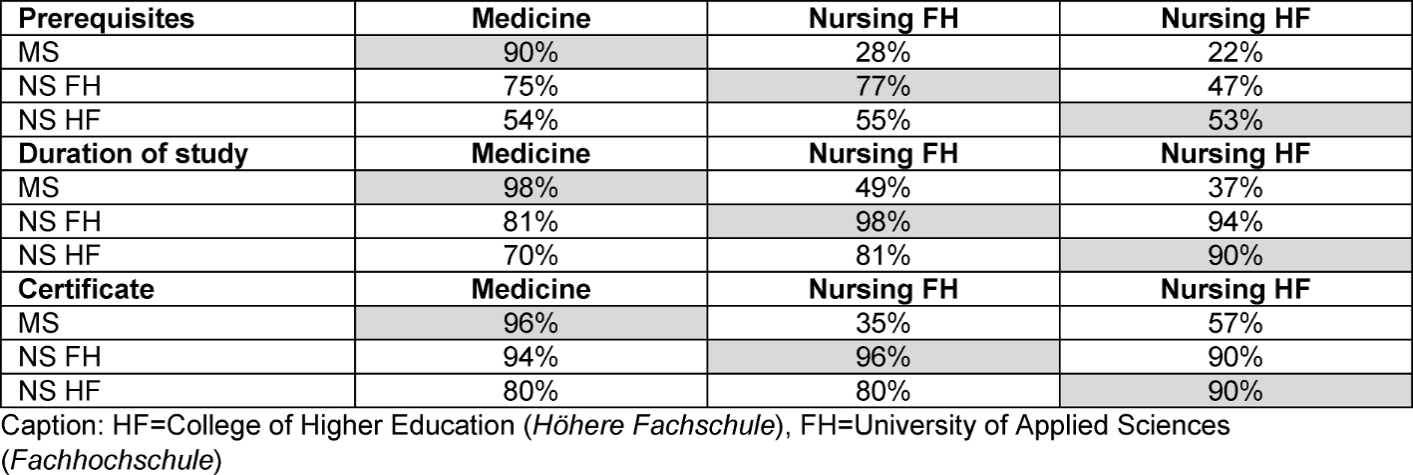
Knowledge about trainings (proper study with grey background)

**Table 3:**
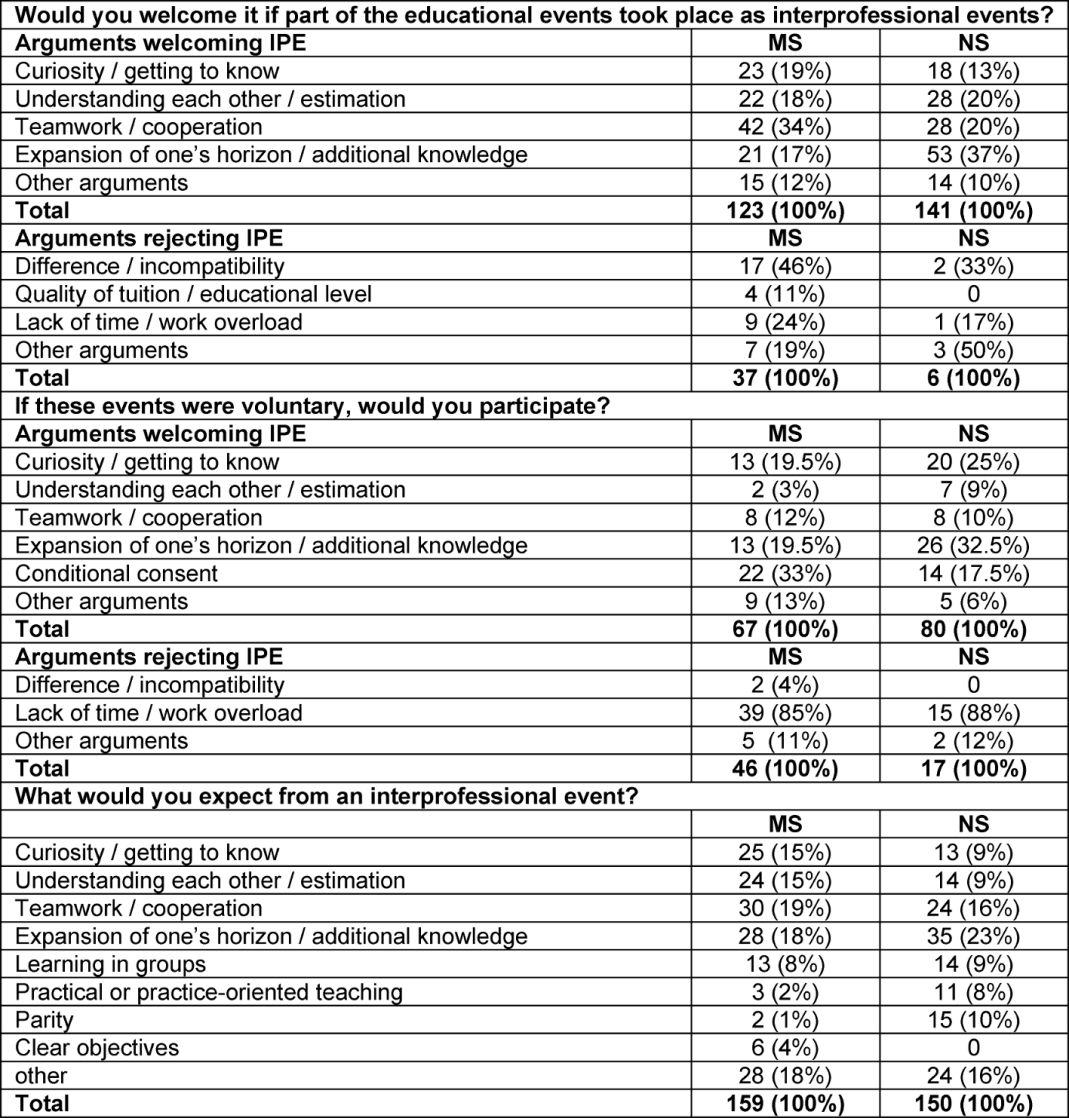
Frequency of arguments in free comments
