# Supplementary material for: Attitude towards and Readiness for Interprofessional Education in Medical and Nursing Students of Bern
Source: GMS J Med Educ. 2016 Nov 15;33(5):Doc73. doi: 10.3205/zma001072 (PMC5135421; doi:10.3205/zma001072)
Supplement: Attachment 1: Assessment of relevance of RIPLS and IEPS items [file JME-33-73-s-001.pdf]

a) Readiness for Interprofessional Learning Scale RIPLS		
**Original / our numbering**	**English original text and German translation *(italics)***	**Relevance mean value**
Item 1 / Item 1	Learning with other students will help me become a more effective member of a health care team.<br>*Gemeinsames Lernen mit Studierenden anderer Gesundheitsberufe wird mir helfen, später ein besseres Mitglied eines Behandlungsteams in der Gesundheitsversorgung zu werden.*	1.1
Item 2 / Item 2	Patients would ultimately benefit if health care students worked together to solve patient problems.<br>*Letztlich würden die Patienten davon profitieren, wenn Studierende verschiedener Gesundheitsberufe zusammenarbeiten würden, um Patientenprobleme zu lösen.*	1.3
Item 3 / Item 3	Shared learning with other health care students will increase my ability to understand clinical problems.<br>*Gemeinsames Lernen mit Studierenden anderer Gesundheitsberufe wird meine Fähigkeit, klinische Probleme zu verstehen, verbessern.*	2.0
Item 4 / Item 4	Learning with health care students before qualification would improve relationships after qualification.<br>*Gemeinsames Lernen mit Studierenden anderer Gesundheitsberufe während der Ausbildung würde die späteren Beziehungen im Berufsalltag verbessern.*	2.0
Item 5	Communication skills should be learned with other health care students.	2.3
Item 6 / Item 5	Shared learning will help me to think positively about other professionals.<br>*Gemeinsames Lernen mit Studierenden anderer Gesundheitsberufe wird mir helfen, positiv über diese Berufsgruppen zu denken.*	2.0
Item 7	For small group learning to work, students need to trust and respect each other.	2.1
Item 8 / Item 6	Team-working skills are essential for all health care students to learn.<br>*Das Erlernen von Team-Working-Fertigkeiten ist für alle Studierenden in Gesundheitsberufen unerlässlich.*	1.6
Item 9	Shared learning will help me to understand my own limitations.	2.1
Item 10	I don’t want to waste my time learning with other health care students.	2.3
Item 11	It is not necessary for undergraduate health care students to learn together.	2.1
Item 12	Clinical problem-solving skills can only be learned with students from my own department.	2.1
Item 13 / Item 7	Shared learning with other health care students will help me to better communicate with patients and other professionals.<br>*Gemeinsames Lernen mit Studierenden anderer Gesundheitsberufe wird mir helfen, besser mit Patienten und anderen Berufsgruppen zu kommunizieren.*	1.6
Item 14	I would welcome the opportunity to work on small-group projects with other health care students.	2.1
Item 15 / Item 8	Shared learning will help to clarify the nature of patient problems.<br>*Gemeinsames Lernen wird dazu beitragen, die Beschaffenheit der Patientenprobleme besser zu verstehen.*	1.7
Item 16 / Item 9	Shared learning before qualification will help me become a better team worker.<br>*Gemeinsames Lernen mit Studierenden anderer Gesundheitsberufe während der Ausbildung wird mir helfen, ein besseres Teammitglied zu werden.*	2.0
Item 17	The function of nurses and therapists is mainly to provide support for doctors.	2.9
Item 18	I'm not sure what my professional role will be.	2.6
Item 19	I have to acquire much more knowledge and skills than other health care students.	3.3

b) Interdisciplinary Education Perception Scale IEPS		
**Original / our numbering**	**English original text and German translation *(italics)***	**Relevance mean value**
Item 1 / Item 1	Individuals in my profession are well-trained.<br>*Personen in meinem Beruf sind gut ausgebildet.*	2.0
Item 2 / Item 2	Individuals in my profession are able to work closely with individuals in other professions.<br>*Personen in meinem Beruf sind in der Lage, eng mit Personen anderer Berufsgruppen zusammen zu arbeiten.*	1.3
Item 3 / Item 3	Individuals in my profession demonstrate a great deal of autonomy.<br>*Personen in meinem Beruf sind in hohem Masse autonom.*	1.9
Item 4 / Item 4	Individuals in other professions respect the work done by my profession.<br>*Personen anderer Berufsgruppen respektieren die Arbeit meiner Berufsgruppe.*	1.6
Item 5	Individuals in my profession are very positive about their goals and objectives.	2.1
Item 6 / Item 5	Individuals in my profession need to cooperate with other professions.<br>*Personen in meinem Beruf müssen mit anderen Berufsgruppen zusammen arbeiten.*	1.7
Item 7	Individuals in my profession are very positive about their contributions and accomplishments.	2.1
Item 8 / Item 6	Individuals in my profession must depend upon the work of people in other professions.<br>*Personen in meinem Beruf müssen sich auf die Arbeit von Personen anderer Berufsgruppen verlassen können.*	1.7
Item 9	Individuals in other professions think highly of my profession.	2.3
Item 10 / Item 7	Individuals in my profession trust each other's professional judgment.<br>*Personen in meinem Beruf trauen dem fachlichen Urteil ihrer Kollegen/innen.*	2.0
Item 11	Individuals in my profession have a higher status than individuals in other professions.	2.3
Item 12 / Item 8	Individuals in my profession make every effort to understand the capabilities and contributions of other professions.<br>*Personen in meinem Beruf bemühen sich sehr, die Fähigkeiten und Beiträge anderer Berufsgruppen zu verstehen.*	1.9
Item 13	Individuals in my profession are extremely competent.	2.9
Item 14 / Item 9	Individuals in my profession are willing to share information and resources with other professionals.<br>*Personen in meinem Beruf sind bereit Informationen und Ressourcen mit anderen Berufsgruppen zu teilen.*	1.6
Item 15 / Item 10	Individuals in my profession have good relations with people in other professions.<br>*Personen in meinem Beruf haben gute Beziehungen zu Personen aus anderen Berufen.*	1.6
Item 16 / Item 11	Individuals in my profession think highly of other related professions.<br>*Personen in meinem Beruf halten viel von Personen von anderen Gesundheitsberufen.*	1.9
Item 17 / Item 12	Individuals in my profession work well with each other.<br>*Personen in meinem Beruf arbeiten gut miteinander zusammen.*	2.0
Item 18 / Item 13	Individuals in other professions often seek the advice of people in my profession.<br>*Personen aus anderen Berufen holen oft Rat von Personen in meinem Beruf.*	1.9

Caption: English original text of RIPLS and IEPS, respectively. German translation (*italics*) of items considered relevant (limit at mean value≤2 based on Likert scale of 1=very relevant, to 6=not at all relevant).
