# Supplementary material for: Attitude towards and Readiness for Interprofessional Education in Medical and Nursing Students of Bern
Source: GMS J Med Educ. 2016 Nov 15;33(5):Doc73. doi: 10.3205/zma001072 (PMC5135421; doi:10.3205/zma001072)
Supplement: Attachment 2: Prerequisites, duration, and certificate of 14 different studies/trainings [file JME-33-73-s-002.pdf]

**Occupation**	**Prerequisites**	**Duration**	**Certificate**
Physician	University-entrance diploma plus aptitude test *(gymnasiale Matur und Eignungstest)*	6 years	Master of Medicine
Assistent in health and social issues *(Assistent/in Gesundheit und Soziales AGS)*	Completion of compulsory schooling	2 years	Federal vocational certificate *(Eidgenössisches Berufsattest)*
Occupational therapist FH	Federal certificate of competency plus professional school leaving examination, or university-entrance diploma, or technical school leaving examination *(Eidgenössisches Fähigkeitszeugnis mit Berufsmatura oder gymnasiale Matura oder Fachmatura)*	3 years	BSc FH
Nutritionist FH	Federal certificate of competency plus professional school leaving examination, or university-entrance diploma, or technical school leaving examination *(Eidgenössisches Fähigkeitszeugnis mit Berufsmatura oder gymnasiale Matura oder Fachmatura)*	3 years	BSc FH
Surgical techniques specialist *Fachfrau/Fachmann Operationstechnik HF*	Federal certificate of competency, or university-entrance diploma, or technical school leaving examination *(Eidgenössisches Fähigkeitszeugnis oder gymnasiale Matura oder Fachmatura)*	3 years	Diploma HF
Health specialist FaGe *(Fachperson Gesundheit FaGe)*	Completed compulsory schooling	3 years	Federal certificate of competency *(Eidgenössisches Fähigkeitszeugnis)*
Medical-technical radiology specialist HF *(Fachperson für medizinisch-technische Radiologie HF)*	Federal certificate of competency, or university-entrance diploma, or technical school leaving examination *(Eidgenössisches Fähigkeitszeugnis oder gymnasiale Matura oder Fachmatura)*	3 years	Diploma HF
Midwife / maternity helper FH	Federal certificate of competency plus professional school leaving examination, or university-entrance diploma, or technical school leaving examination *(Eidgenössisches Fähigkeitszeugnis mit Berufsmatura oder gymnasiale Matura oder Fachmatura)*	3 years	BSc FH
Medical practice assistant	Completed compulsory schooling	3 years	Federal certificate of competency *(Eidgenössisches Fähigkeitszeugnis)*
Nurse HF *(Pflegefachfrau/-mann HF)*	Federal certificate of competency, or university-entrance diploma, or technical school leaving examination *(Eidgenössisches Fähigkeitszeugnis mit Berufsmatura oder gymnasiale Matura oder Fachmatura)*	3 years	Diploma HF
Nurse FH *(Pflegefachfrau/-mann FH)*	Federal certificate of competency plus professional school leaving examination, or university-entrance diploma, or technical school leaving examination	3 years	BSc FH

	(Eidgenössisches Fähigkeitszeugnis mit Berufsmatura oder gymnasiale Matura oder Fachmatura)		
Physiotherapist FH	Federal certificate of competency plus professional school leaving examination, or university-entrance diploma, or technical school leaving examination *(Eidgenössisches Fähigkeitszeugnis mit Berufsmatura oder gymnasiale Matura oder Fachmatura)*	3 years	BSc FH
Paramedic HF	Federal certificate of competency, or university-entrance diploma, or technical school leaving examination *(Eidgenössisches Fähigkeitszeugnis oder gymnasiale Matura oder Fachmatura)*	3 years	Diplom HF
Dentist	University-entrance diploma plus aptitude test *(gymnasiale Matur und Eignungstest)*	5 years	Master of Dental Medicine

Caption: HF=College of Higher Education (*Höhere Fachschule*), FH=University of Applied Sciences (*Fachhochschule*), BSc=Bachelor of Science
